# Culturing and transcriptome profiling of progenitor-like colonies derived from adult mouse pancreas

**DOI:** 10.1186/s13287-017-0626-y

**Published:** 2017-07-26

**Authors:** Dongshen Ma, Shanshan Tang, Jing Song, Qiong Wu, Fangfang Zhang, Yun Xing, Yi Pan, Yanfeng Zhang, Jingwei Jiang, Yubin Zhang, Liang Jin

**Affiliations:** State Key Laboratory of Natural Medicines, Jiangsu Key Laboratory of Drug Screening, School of Life Science and Technology, China Pharmaceutical University, 24 Tongjiaxiang, Nanjing, Jiangsu People’s Republic of China

**Keywords:** Pancreatic progenitors, 3D culturing system, High-throughput sequencing, MicroRNAs, Long noncoding RNAs

## Abstract

**Background:**

Transplantation of insulin-producing cells is considered an important diabetes therapy. Many research studies have shown that insulin-producing cells can be derived from the in-vitro cultured pancreatic colonies with self-renewal ability and multilineage potential. Even though these progenitor-like colonies have been prepared from adult pancreas cells, the efficient culture method is hardly established and regulation of the colonies is rarely known. We confirmed previously that single cells acquired from adult mouse pancreas could form cyst-like colonies in a 3D semi-solid system containing Matrigel and methylcellulose. These colonies could be passaged continuously without losing progenitor-like capacity. In the previous culturing system, however, conditioned medium from murine embryonic-stem-cell-derived pancreatic-like cells was used. This unregulated ingredient may reduce repeatability and affect following study. Thus, a new culturing system with certain components needs to be developed.

**Methods:**

Single cell suspension was acquired from adult mouse pancreas and cultured in a Matrigel-based 3D system with epidermal growth factor, Nicotinamide, B27, and Noggin to form ring colonies. Serial-passage assay was performed to evaluate self-renewal ability. Real-time polymerase chain reaction and immunostaining were used to detect the expression of progenitor-related genes. A 2D differentiation method was used to testify the multilineage potency of the colonies. High-throughput sequencing (HTS) of the colonies was performed to profile the differentially expressed genes.

**Results:**

We developed a 3D culturing system deprived of conditioned medium to propagate those colonies with high proliferative efficiency. HTS of the transcriptome of mRNAs, microRNAs (miRNAs) and long noncoding RNAs (lncRNAs) showed differentially expressed genes compared to the whole pancreas (as control). In mRNAs, several surface marker genes were identified in the colonies. Moreover in noncoding RNAs, miR-21a, miR-31 and miR-155 were upregulated and miR-217, miR-802 and miR-375 were downregulated in colonies along with a number of other miRNAs and lncRNAs.

**Conclusions:**

Our results offer an efficient culture system for pancreatic progenitor-like colonies and HTS of the colonies serves as a target resource for following study of in-vitro cultured pancreatic progenitors. These findings should also contribute to our understanding of the transcriptional regulation of these progenitor-like colonies and the mechanisms behind their functions.

**Electronic supplementary material:**

The online version of this article (doi:10.1186/s13287-017-0626-y) contains supplementary material, which is available to authorized users.

## Background

Having been known for decades, adult pancreatic cells hold the promising prospect of being used for generating insulin-producing cells [1, 2]. These progenitor-like cells were believed to be located in the duct, centroacina or acinar in various reports [3–5]. Among these, ductal epithelium is widely recognized as a potential pool in vitro and in vivo [6]. These cells remain quiescent in the adult, but when the pancreas is damaged or given certain substances they start to proliferate and then give rise to other pancreatic lineages including beta cells [7, 8]. These cells can differentiate into insulin-producing cells under specific conditions in vitro [9] or be directly transplanted under the kidney capsule for differention ​in-vivo [10]. Hence, an in-vitro culturing system serves as a pivotal tool to enrich them for transplantation.

Previously, we found that CD133^+^/SOX9^high^ cells in adult mouse pancreas could form cyst-like colonies in a 3D culture system [11]. These "ring" colonies maintain self-renewal capacity after several passages and can form endocrine/acinar colonies in laminin hydrogel. Given these facts, this system provides a good platform to research the regulation of the colonies’ functions.

CD133 is a common marker for pancreatic ductal marker [12, 13]. Sex determining region Y-box 9 (SOX9) is a transcriptional factor which plays an important role in pancreas development and is restricted mainly to the duct in adult mice [14]. Our previous study sorted out a CD133^+^/SOX9^high^ subpopulation to form progenitor-like colonies. However, this fraction may not represent all ductal epithelial cells which have colony-forming ability. Moreover, conditioned medium from murine embryonic stem cells has been applied in this culture system, containing uncontrollable components which may hinder the revealing of regulatory factors [11]. For the following analysis of the colonies’ regulation, this prompted us to modify our previous culturing system by propagating single cells from the whole pancreas instead of interrogating only a subpopulation based on limited markers.

In our modified system, single cells from adult mouse pancreas can form PDX1^+^ colonies resembling the previous ones [13]. Pancreatic and duodenal homeobox 1 (PDX1) is a classic marker for pancreas specification and is expressed in ductal epithelium during development, which gives rise to all pancreatic lineages [15]. Duct-originated progenitors derived from C57BL6 mouse regained the ability to express PDX1 if cultured in vitro, which could explain the pluripotency of these cultured progenitors [11, 13]. During culture, PDX1 and proliferation marker Ki67 are expressed continuously in our colonies with stable ductal epithelium phenotype, indicating that they are proper materials for further investigation.

To accumulate more colonies for beta-cell generation or transplantation, the regulation of their functions (especially proliferation and differentiation) should be examined carefully to find vital regulators. Therefore, we performed high-throughput sequencing (HTS) of the transcriptome of the colonies versus whole pancreas to find specific RNAs in the colonies. HTS, also named “next generation sequencing”, is extensively used to profile RNA transcriptome in cancer stem cells [16] and adult stem cells [17]. With this technology, coding mRNAs and noncoding RNAs (microRNAs (miRNAs) and long noncoding RNAs (lncRNAs)) can be quantified accurately and efficiently, making it a great help in development and stem/progenitor cell study [18].

In addition to illustrating the properties of specific cells, HTS can also provide a large amount of valuable information such as new surface markers. Surface markers are surface antigens located within the cell membrane. They can be detected easily by flow cytometry (FCM), making the cells sortable without gene modification [19]. Discovery of new surface markers and sorting for new subpopulations of pancreatic progenitor-like colonies can help the enrichment of potential cells with high colony-forming efficiency. Some of the specific markers have been discovered, including our previously reported CD71 [20]. In this study we recorded 7266 mRNA changes in our colonies compared to pancreas. By analyzing mRNAs, we captured significant changes in gene expressing profile including surface antigens which could be used as surface markers for cell sorting.

Along with mRNAs, 285 miRNA and 183 lncRNA changes were detected. miRNAs are small noncoding RNAs which can bind target mRNAs to silence their expressions [21]. miRNAs have critical effects on the pancreatic differentiation from pluripotent stem cells [22] and beta-cell functions [23]. Our previous study has shown that miR-26a is regulated in differentiation by targeting ten–eleven translocation (TET) enzymes [24]. lncRNAs are also noncoding RNAs discovered recently. They regulate gene expression through many mechanisms [25]. There have been plenty of studies on lncRNAs regulating pancreatic cancer [26], beta-cell biology [27] and pancreas development [28]. However, neither miRNAs nor lncRNAs are deeply researched in regulation of pancreatic progenitor-like colonies.

Our study for the first time profiled the transcriptome of pancreatic progenitor-like colonies. Compared to transcriptome of whole pancreas, we found highly upregulated mRNAs, miRNAs and lncRNAs along with potential surface markers. With Gene Ontology (GO) and Kyoto Encyclopedia of Genes and Genomes (KEGG) analysis, these findings should help the understanding of these colonies and the regulatory network of proliferation and differentiation.

## Methods

### Animals

C57BL/6 J mice (6–8 weeks old) were obtained from the Model Animal Research Center of Nanjing University (Nanjing, China). All care and handling of animals were carried out according to the international laws and policies (EEC Council Directive 86/609, 1987) and approved by the animal ethics committee of China Pharmaceutical University (Nanjing, China) (Reference Number: 2162326).

### Single cell preparation and culturing

For 3D culture, pancreas from 8-week-old C57BL/6 J mouse was isolated and cut into 1-mm^3^ pieces on a cold dish, followed by 30-min digestion in 2 mg/ml collagenase II at 37 °C to obtain single cells. Then 8000 live cells were counted after trypan blue staining and were seeded into a 24-well ultra-low attachment plate (Corning) in DMEM/F12 (Gibco) supplied with 10% Matrigel (Corning), 1% methylcellulose (1500 cP; Sigma), 20 ng/ml EGF (Sigma), 10 mM Nicotinamide (Sigma), 100 ng/ml Noggin, 2% B27 (Invitrogen), 5% FBS (Gibco), 100 U/ml penicillin and 100 μg/ml streptomycin (Gibco).

For 2D culture, ring colonies were dissolved in 0.25% trypsin to obtain single cells. Cells were counted and seeded in Matrigel-coated (10%) plates in DMEM/F12 supplied with 5 μM SB431542 (Sigma), 20 ng/ml EGF, 10 mM Nicotinamide (Sigma), 1% B27, 5% fetal bovine serum (FBS), 100 U/ml penicillin and 100 μg/ml streptomycin (Gibco).

For beta-lineage differentiation, the method reported previously was employed [29]. Cells were cultured in a 2D system with serum-free DMEM/F12 supplemented with ITS (5 mg/l insulin, 5 mg/l transferrin. 5 μg/l sodium selenite; Gibco), 2 g/l bovine serum albumin (BSA; Biosharp), 8 mmol/l glucose (Sigma), 10 mmol/l nicotinamide, 100 U/ml penicillin and 100 μg/ml streptomycin (Gibco). After the medium was changed, 10% Matrigel was applied on the top of the cells with overnight gelling at 37 °C.

Colonies were cultured in 37 °C with 5% CO_2_ for 2–4 weeks depending on the experiments.

### Serial passaging assay

For 3D culture, after 2 weeks of culture all of the colonies were counted and hand-picked under an inverted microscope. After dissolving in 0.25% trypsin, cells were counted and replated in the same condition. This process was repeated five times.

### RNA extraction

For the pancreas, adult mice were sacrificed and dissected immediately to isolate the pancreas. Fresh pancreas was cut into 3-mm^3^ pieces and immersed in RNAlater (Qiagen) for 1 h at room temperature before extraction. For ring colonies, single colonies were hand-picked using a 20-μl pipette under an inverted microscope and collected in a 1.5-ml tube. For adherent cells, QiAzol Lysis Reagent (Qiagen) was directly added to the cells to obtain cell lysate. All samples were then homogenated and RNA was extracted using the Qiagen RNeasy Mini kit (Qiagen).

### Real-time PCR and RT-PCR

The real-time PCR and RT-PCR were performed following standard procedures. cDNA was prepared using either All-In-One RT MasterMix (ABM) or the specific miRNA/lncRNA reverse transcription kit (Genepharma). Real-time PCR was performed using EvaGreen 2X qPCR MasterMix (ABM) on a Light Cycler 480 II (Roche). RT-PCR was performed using the EasyScript Two-Step RT-PCR Kit (ABM) on a normal PCR instrument (Thermo). The RT-PCR products were separated and detected using 1.5% agarose (Lonza). The primers used in RT-PCR and real-time PCR are presented in Additional file 1: Table S1.

### Immunostaining

Colonies were hand-picked using a 20-μl pipette, fixed in 4% paraformaldehyde (Sigma) for 10 min at room temperature and blocked with Blocking Buffer (Abcam) at 37 °C for 1 h, followed by primary antibody incubation with PDX1 (1:500), Ki67 (1:200), cytokeratin 7 (CK7; 1:250), neurogenin 3 (NEUROG3; 1:50), SOX9 (1:200), or C-peptide (1:250). The colonies were finally detected with secondary antibodies conjugated to Alexaflour 488 or Alexaflour 650 (Abcam) at a dilution of 1:250. Images were captured by a laser scanning confocal microscopy (Olympus) or an inverted fluorescence microscope (Olympus).

### Flow cytometry and cell sorting

For the pancreas, single cells were prepared as already mentioned. For colonies, single colonies were hand-picked and digested in trypsin. Cells were blocked using CD16/32 antibody (eBioscience) and stained with CD133-APC antibody (eBioscience) or its isotype anti-Rat IgG1 (eBioscience). After washing twice in PBS, the CD133^+^ cells were detected by Accuri C6 (BD) or sorted by Aria (BD).

### High-throughput sequencing

Three independent samples of colonies and their preculture controls (pancreas) were harvested for HTS. RNA was extracted as already mentioned. An RNA library was constructed using the TruSeq RNA LT Sample Prep Kit. Then the cluster generation process was performed using the TruSeq PE Cluster Kit on a cBot system. The sequencing process was performed using the TruSeq SBS Kit on a Hiseq2500. All of the kits and instruments were obtained from Illumina. Also, Newbler2.7 was used for data analysis and Mus musculus GRCm38.p5 (Ensemble) was used for comparison.

### Statistical analysis

Data are presented as the mean ± SD. Two-tailed *t* test was used to assess the differences between experimental groups. Statistical significance was defined as *p* < 0.05, *p* < 0.01 and *p* < 0.005.

## Results

### Dissociated mice pancreas forms cyst-like ring colonies in modified 3D medium

**Fig. 1:**
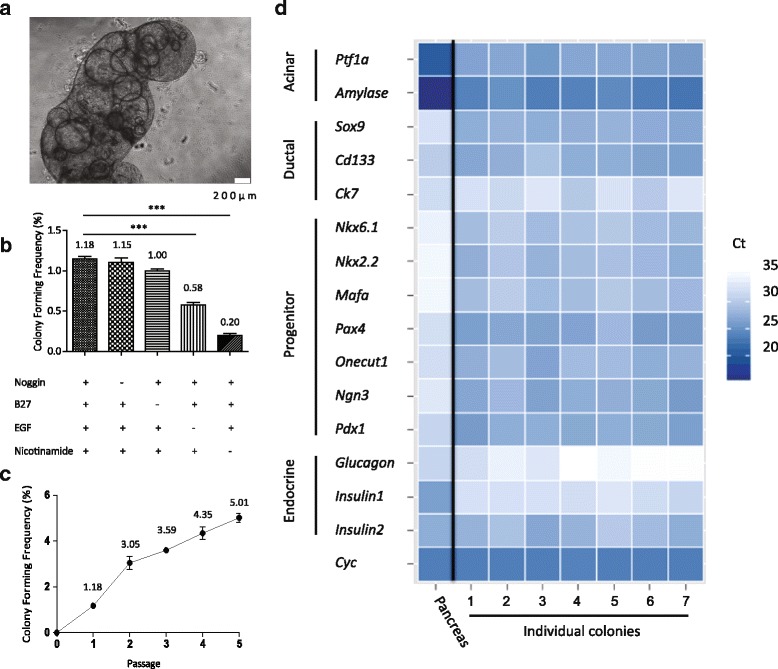
Single cells from adult mouse pancreas could form ring colonies. **a** Single cells from adult mouse pancreas formed ring colonies after 2 weeks of culture in 3D medium. *Scale bars*, 200 μm. **b** Colony-forming frequency with/without certain factors in medium. EGF and Nicotinamide were pivotal for colony-forming. **c** Self-renewal capacity tested by serial passaging. **d** Pancreas-related genes analyzed by real-time PCR. Two-week-old ring colonies (*n* = 7) enriched pancreatic ductal and progenitor genes. ***p < 0.005. *EGF* epidermal growth factor, *Sox9* sex determining region Y-box 9, *Ck7* cytokeratin 7, *Ngn3* Neurog 3, *Ptf1a* pancreas specific transcription factor 1α, *Nkx6.1* NK6 homeobox 1, *Nkx2.2* NK2 homeobox 2, *Mafa* v-maf musculoaponeurotic fibrosarcoma oncogene family, protein A, *Pax4* paired box 4, *Cyc* cyclophilin (Color figure online)

To replace the conditioned medium from murine embryonic stem cells used in our previous 3D culture system, new ingredients were applied and the concentration of Matrigel was elevated to 10%. After culturing for 2 weeks (Additional File 2: Figure S1A), organoid-like ring colonies formed in concentrated Matrigel (Fig. 1a) with a colony-forming efficiency of about 1.2% (Additional file 3: Table S2), resembling the published data [13]. The diameters of most colonies were under 500 μm (Additional File 2: Figure S1B). The cell numbers of different colonies are represented in Additional File 2: Figure S1C with a fitted curve.

We knocked out each of the components in our medium (except FBS) to interrogate its impacts on colony-forming frequency. Of note, EGF and Nicotinamide are pivotal for colony-forming while Noggin and B27 exhibited slight effects (Fig. 1b). Also, DMEM/F12 showed more efficiency than H-DMEM and L-DMEM (Additional File 2: Figure S1D).

Self-renewal is one of the properties of pancreatic progenitors and allows them to be passaged continuously in vitro. Thus, a serial passaging method was performed to evaluate the self-renewal ability of our colonies. Two-week-old colonies were hand-picked and trypsinized. The cells were then replated in new medium until secondary ring colonies emerged. As shown in Fig. 1c, the colony-forming frequency increased along with the cell passage but the colony size was smaller after subculture for five passages (Additional File 2: Figure S1E). The results indicated that colony-forming cells were enriched in this culture system and they did not lose the capacity of self-renewal when subcultured at least within five passages, while somehow its proliferation ability declined.

To assess the mRNA expression pattern of the colonies, we used real-time PCR to test a sum of pancreatic specific genes or those relating to pancreas development. Two-week-old ring colonies were picked individually and dissolved for RNA extraction. Nearly all of the pancreatic specific genes were detected in ring colonies (Fig. 1d). Compared to pancreas, the colonies highly expressed ductal marker *Sox9*, *CD133*, *Ck7* and other endocrine-relevant genes in pancreas development such as NK6 homeobox 1 (*Nkx6.1*), NK2 homeobox 2 (*Nkx2.2*), paired box 4 (*Pax4*), *Neurog3*, etc. Detected acinar and mature endocrine transcripts in the colony indicated pluripotency when cultured in vitro.

### Ring colonies express pancreatic progenitor markers confirmed by immunostaining

**Fig. 2:**
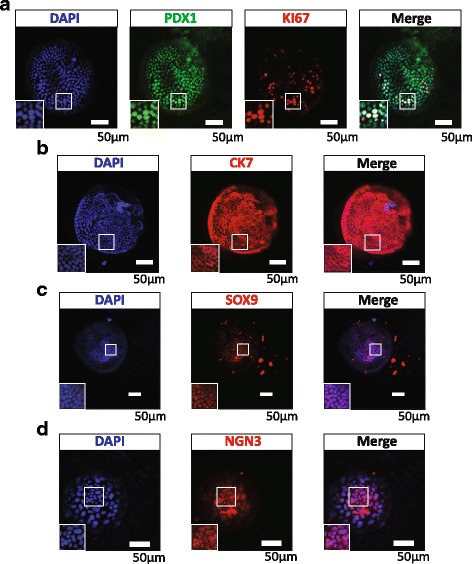
Immunostaining of pancreatic progenitor-related transcriptional factors in ring colonies. **a**–**d** Whole-mount immunostaining of single ring colonies showed PDX1/Ki67, CK7, SOX9 and NEUROG3 expression. *Scale bars*, 50 μm. PDX1/Ki67 coexpression indicated a proliferative pancreatic progenitor-like phenotype. CK7 and SOX9 showed the ductal origin of the colony. Weak expression of NEUROG3 implied its potential for endocrine lineage. *SOX9* sex determining region Y-box 9, *CK7* cytokeratin 7, *NGN3* neurog 3, *PDX1* Pancreatic and duodenal homeobox 1 (Color figure online)

Transcriptional factors which are critical in pancreas development such as PDX1 reoccur in cultured pancreatic progenitors [8]. Our data showed extensive PDX1 expression in ring colonies along with proliferation marker Ki67 (Fig. 2a). Expression of CK7 and SOX9 confirmed its ductal origin (Fig. 2b, c). Endocrine progenitor marker NEUROG3 was also detected (Fig. 2d). Although weak, the presence of NEUROG3 suggested the potential to generate endocrine lineages. Interestingly, after 3 weeks of culture, some of the colonies (~10%, especially in large colonies) became shriveled and compact (Additional File 4: Figure S2). C-peptide, a marker for de-novo synthesized insulin, was detected in the cytoplasm (Additional File 4: Figure S2), demonstrating obvious endocrine commitment. These results indicated that, in our modified 3D culture system, ring colonies showed a progenitor-like phenotype and gene expression pattern. Moreover, with Ki67 expressed widely, our system offered a proper environment for proliferation.

### Cells in the colonies can be expanded as 2D plaques and form C-peptide secreting clusters

**Fig. 3:**
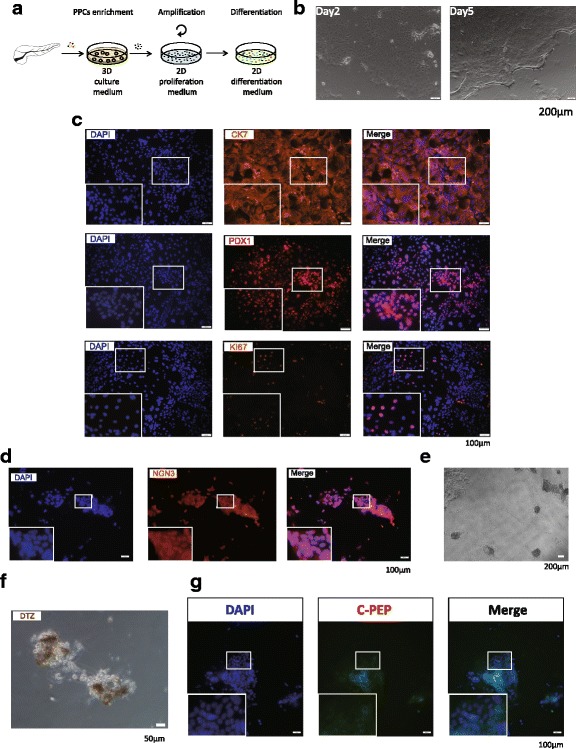
Cells from ring colonies can be induced to differentiate to beta lineage. **a** After enrichment in a 3D system, colonies after three passages were dissolved and plated in a 2D system for differentiation. *Scale bars*, 200 μm. **b** Plaques formed in a 2D system and cells reached nearly 100% confluence in 5 days. **c** Immunostaining showing expression of CK7, PDX1 and Ki67 in 2D plaques. *Scale bars*, 100 μm. **d** NEUROG3 detected after 1 week of culture in differentiation medium, indicating the commitment of endocrine progenitors. *Scale bars*, 200 μm. **e**–**g** Clusters found at day 14 in differentiation medium and the staining of DTZ and C-peptide was positive. *Scale bars*, 50 μm. *C-pep* C-peptide, *CK7* cytokeratin 7, *DTZ* Dithizone, *PDX1* Pancreatic and duodenal homeobox 1 (Color figure online)

To further assess the multilineage potency of our cultured colonies, we applied a method published previously to induce pancreatic progenitors to beta lineage [29]. Before differentiation, the cells in our colonies have to be plated into the 2D system. We employed 2D medium used to culture embryo stem cell-derived pancreatic progenitors (Fig. 3a). As expected, after dissociation from ring colonies and seeding in a Matrigel-coated plate, the cells (after three passages in a 3D system as colonies) formed epithelial plaques in 24–48 h (Fig. 3b). After 5 days of culture, cells were grown into a confluent monolayer (Fig. 3b). We passaged the cells in a 1:2–1:3 ratio every 5 days at least five times without an observed shape change (data not shown).

Confirmed by immunostaining, 2D plaques recapitulated the expression of CK7, PDX1 and Ki67 in ring colonies (Fig. 3c). The differentiation method was applied to induce the colony-derived cells to beta lineage after the plaques reached 100% confluence. Most of the cells went through apoptosis after being plated over 1 week, with NEUROG3 being broadly detected (Fig. 3d). Two weeks later, small dense clusters appeared (Fig. 3e) at a frequency of about 1.5 colonies per 10,000 cells. The presence of insulin in the clusters was confirmed by Dithizone (DTZ) staining (Fig. 3f). C-peptide was detected, indicating the forming of beta-cell-like clusters (Fig. 3 g). Besides, exocrine genes *Amylase*, *Somatostatin* and chymotrypsin-like elastase (*Cela1*) and endocrine genes *Insulin1* and *Insulin2* were confirmed to be expressed in some of the clusters after induction (Additional File 5: Figure S3), which signified the pluripotency of the cells in the colonies.

### High-throughput sequencing and transcriptome profiling of the colonies

**Fig. 4:**
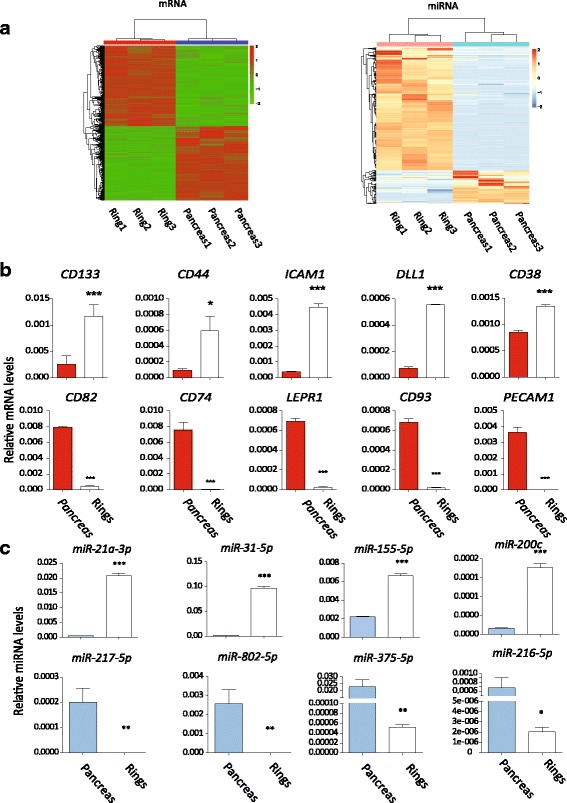
Differentially expressed mRNAs and miRNAs detected by HTS and verified by real-time PCR. **a** Heat map of all mRNAs and miNRAs showing differences in the transcriptome of the colonies and control. Three independent samples of colonies and their preculture controls (pancreas) were harvested for HTS. **b**, **c** Verification of differentially expressed mRNAs and miRNAs by real-time PCR. *p < 0.05, **p < 0.01 and ***p < 0.005, comparison to the control group. CD133, CD44, ICAM1, DLL1 and CD38 were enriched in ring colonies. miR-21a-3p, miR-31-5p, miR-155-5p and miR-200c were upregulated while miR-217-5p, miR-802-5p, miR-375-5p and miR-216-5p were downregulated (Color figure online)

We performed HTS to illuminate the gene expression pattern of our colonies and to discover the regulation factors. RNA of hand-picked colonies and whole pancreas (as control) was extracted, purified and sequenced. Compared to the pancreas, 7266 mRNAs, 285 miRNAs (111 previously unidentified) and 183 lncRNAs (23 previously unidentified) were found differentially expressed in colonies (Fig. 4a, Additional File 6: Figure S4A and Additional File 7: Table S3). The top 25 differentially expressed mRNAs, miRNAs and lcnRNAs were listed (Additional file 8: Table S4, Additional file 9: Table S5 and Additional file 10: Table S6). The original datasets for HTS are also provided (Additional file 11: Table S7 and Additional file 12: Table S8).

Surprisingly, in upregulated mRNAs, it was not the classical pancreatic progenitor-related genes that changed the most. Methyltransferase like 10 (*Mettl10*), a methyltransferase-like gene, took first place of the upregulated genes. Although not functionally characterized, it has been found to specifically trimethylate eukaryotic translation elongation factor 1 alpha 1 (*Ef1a1*), a eukaryotic elongation factor, which implies its contribution to many biological processes on translational level [30]. *Onecut2*, a member of the onecut family, is also among those most upregulated genes. It is known as downstream of paired box 6 (*Pax6*), and least partially contributed to pancreas specification and endocrine differentiation [31, 32]. Another upregulated gene, fibroblast growth factor receptor 2 (*Fghr2*), indicated the important role of FGF signal in regulating of the colonies. Inhibition of *Fghr2* in pancreatic cancer has been shown to impair proliferation [33], implying its proliferation-promoting function on pancreas-originated cells. On the other hand, pancreatic exocrine genes such as trypsin 4 (*Try4*), chymotrypsin (*Ctrl*) and *Insulin1* were among the downregulated genes, indicating the expected differential between colonies and pancreas control.

We focused on some of the transmembrane protein genes of significantly changed mRNAs. These up/downregulated genes could be regarded as a positive/negative surface marker pool for screening. We verified 10 of these genes by real-time PCR (Fig. 4b). Some signal pathway receptors (delta-like 1 (*Dll1*), frizzled class receptor 1 (*Fzd*)), some cell adhesion molecules (intercellular adhesion molecule 1 (*Icam1*) and other clusters of differentiation (CDs) were among them. Especially, CD133, a classic marker for pancreatic stem cells [19], was upregulated in our colonies (~4-fold higher in HTS results).

We also verified eight miRNAs (Fig. 4c). We noted that miR-31-5p, miR-21a-3p/5p, miR-155-5p and miR-29b-3p were upregulated. miR-31 is engaged in regulating the proliferation of pancreatic cancer cells [34], indicating its potential role in regulating pancreatic-originated cells. miR-21a has been reported recently to be an important regulator in pancreatic progenitors in chicken [35]. miR-155 and miR-29b were recently reported upregulated in the pancreas of a transgenetic model mouse for diabetes [36]. In downregulated miRNAs, miR-216a-3p/5p, miR-216b-3p/5p, miR-217-5p, miR-802-3p/5p and miR-375-3p lay on the top. miR-216/miR-217 were supposed to be reduced in pancreatic adenocarcinoma [37]. miR-802 is highly expressed in the liver and pancreas, and regulates insulin resistance by targeting HNF1 homeobox B (HNF1B) in the liver [38], while its function in the pancreas remains unknown. miR-375 is a well-known miRNA in regulating beta-cell development [39] and is used to generating insulin-producing cells from induced pluripotent cells [40]. The reported function of these mostly changed miRNAs is summarized in Table 1. However, none of these miRNAs is deeply studied in pancreatic progenitors. Moreover, eight lncRNAs were also verified (Additional File 6: Figure S4B), including metastasis associated lung adenocarcinoma transcript 1 (*Malat1*), a lncRNA recently shown to promote pancreatic cancer cell proliferation [41]. Little is known about these lncRNAs in pancreas development or functions.

**Table 1:** Reported functions of the most significantly changed miRNAs

miRNA	Up/downregulated	Fold change (log_2_)	Function	Reference
miR-31-5p	Upregulated	10.19	Regulating migration of PDAC	[34]
miR-21a-5p	Upregulated	7.89	Regulating pancreatic progenitors in chicken^a^	[35]
miR-155-5p	Upregulated	7.80	Biomarker in PDAC	[36]
miR-217-5p	Downregulated	–9.10	Reduced in mouse model of PDAC	[37]
miR-802-5p	Downregulated	–6.81	Targeting *HNF1B*	[38]
miR-375-3p^b^	Downregulated	–6.66	Promotes beta pancreatic differentiation in hiPS	[40]

*PDAC* pancreatic ductal adenocarcinoma, *hiPS* human induced pluripotent stem cells

These miRNAs were among the most significantly changed. They have been reported to be important in regulation of either pancreatic progenitors or pancreatic ductal adenocarcinoma

^a^mmu-miR-21a-5p is homologous to gga-miR-21 in chicken

^b^Previous ID of mmu-miR-375-3p is “mmu-miR-375”

### Colonies from whole pancreas are comparable to CD133^+^ cell-derived colonies

**Fig. 5:**
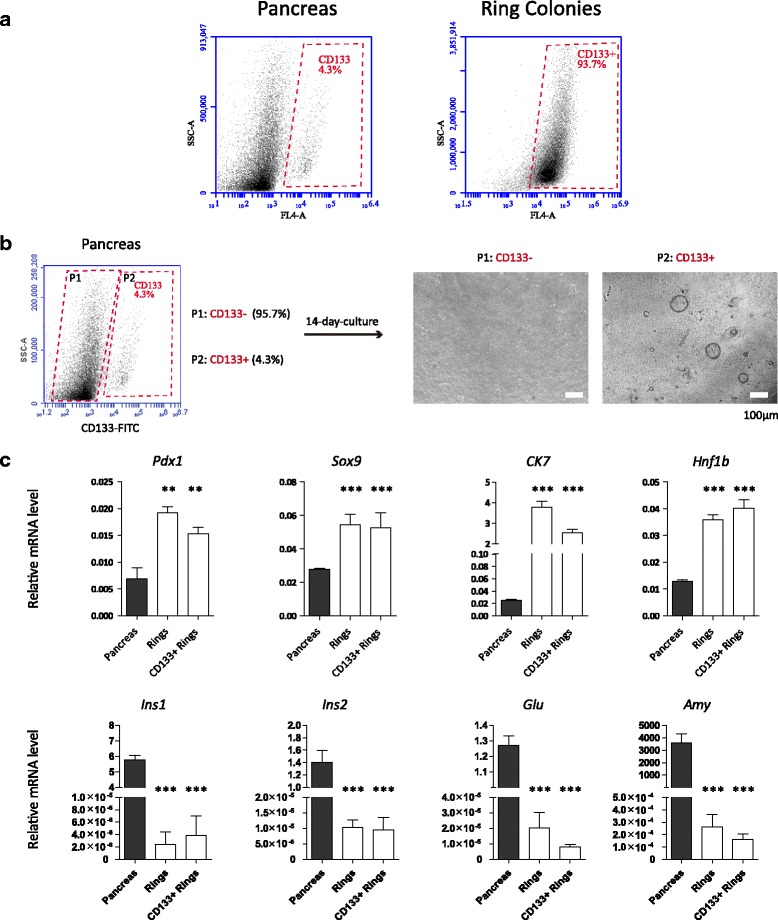
Most cells in colonies were CD133^+^ and the expression pattern of pancreatic progenitor-related genes was comparable to CD133^+^ cell-derived colonies. **a** CD133^+^ cells consist of more than 90% of the colony cells while only less than 5% in pancreas tissue. **b** CD133^+^ and CD133^–^ cells were sorted respectively by BD Aria. Only CD133^+^ cells could form ring colonies in a 3D system. **c** Ring colonies from total pancreas cells and the CD133^+^ subset showed a similar expression pattern. **p < 0.01, ***p < 0.005

CD133 is a well-recognized marker for pancreatic progenitors [11, 12, 19]. HTS results and real-time PCR showed higher expression of CD133 in colonies (Fig. 4b). We then tested the CD133 expression in colonies using FCM. As shown in Fig. 5a, only ~4% cells were CD133^+^ while in the colonies more than 90% of the cells were CD133^+^. CD133^+^ and CD133^–^ cells were sorted respectively through BD Aria and seeded in a 3D system. Surprisingly, only CD133^+^ cells could form "ring" colonies in our system after 2 weeks (Fig. 5b). Real-time PCR confirmed that CD133^+^ cell-derived colonies shared the same gene expression pattern with the colonies from the total pancreas cells (Fig. 5c). These results indicated that the ring colonies should be derived from the CD133^+^ subset of pancreatic cells and could be reliable materials for HTS because all colony-forming subsets were included.

### GO and KEGG enrichment of target genes

GO is a dataset to annotate and classify all differentially expressed genes in order to reveal the most changed components or functions. Each GO category includes many terms (listed by gene counts) into which differentially expressed genes (in this study, colonies versus control) distribute. For noncoding RNA, GO analysis is applied to its target genes. The *p* value is calculated for each GO term to represent the probability that gene counts in such a term could result from random distribution.

**Fig. 6:**
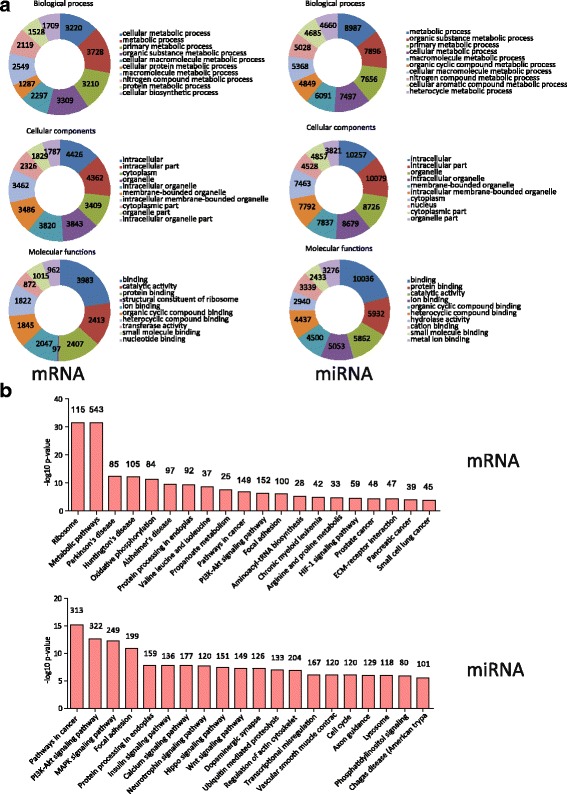
GO and KEGG enrichment of mRNAs and miRNAs. **a** GO enrichment of significantly changed mRNAs (*left*) and miRNAs (*right*). Top 10 (by *p* value) for each GO dataset shown. *Numbers* in each part of the graph stand for the gene counts in each GO dataset. **b** Top 20 (by *p* value) of enriched KEGG pathways of mRNAs and miRNAs. *Numbers* on top of each column stand for the gene counts in each KEGG pathway. *miRNA* microRNA

The top 10 GO terms of differentially expressed genes in the colonies were listed by *p* value (Fig. 6a). In mRNA, differentially expressed genes were enriched in the biological processes of "cellular metabolic progress", "metabolic process" and "primary metabolic process". The cellular component genes were mainly enriched in "intracellular", "intracellular parts" and "cytoplasm". The molecular functions of these genes were "binding", "catalytic activity" and "protein binding". For predicted miRNA targets, differentially expressed genes were enriched in the biological processes of "metabolic process", "organic substance metabolic process" and "primary metabolic process". The cellular component genes were mainly enriched in "intracellular", "intracellular part" and "organelle". The molecular functions of these genes were "binding", "protein binding" and "catalytic activity". For lncRNA target genes, the top GO terms were also listed (Additional File 13: Figure S5A).

KEGG analysis showed the pathways in which differentially expressed genes were enriched. KEGG analysis of the mRNA transcriptome showed that "Ribosome" and "Metabolic pathways" were the most enriched pathways (Fig. 6b, upper). In miRNA target genes, "Pathways in cancer", "PI3K-Akt signaling pathways", "MAPK pathways" and "Focal adhesion" were the most enriched pathways (Fig. 6b, lower). Pathways enriched in lncRNA target genes are shown in Additional File 13: Figure S5B.

### Coexpression analysis of differentially expressed RNAs

One of the mechanisms of miRNAs in regulating mRNAs is through the cleavage of mRNA [21], indicating that the expression trend of a miRNA and its target mRNA is likely to be opposite. Also, a lncRNA can affect the expression of mRNAs through specific mechanisms [25]. Thus the overlapped genes (targets) among significantly changed mRNAs, miRNAs and lncRNAs may play important roles in regulating the network.

**Fig. 7:**
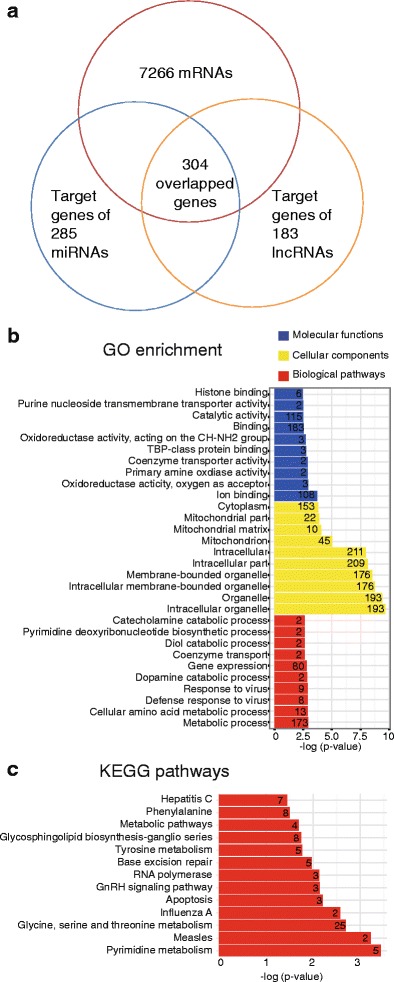
Coexpression analysis of mRNAs, miRNAs and lncRNAs. **a** In total, 304 overlapped genes were found from mRNAs and targets of noncoding RNAs. **b** GO and **c** KEGG enrichment of overlapped genes (listed by *p* value). *Numbers* in each row stand for the gene counts in each GO dataset or KEGG pathway. *GO* Gene Ontology, *miRNA* microRNA, *lncRNA* long noncoding RNA (Color figure online)

To find key mRNAs regulated by the noncoding RNA network, we performed coexpression analysis of differentially expressed RNAs between the colonies and control. The intersection of significantly changed mRNAs and the potential targets of all significantly changed noncoding RNAs were obtained (Fig. 7a). We noted 304 overlapped genes. The top five up/downregulated genes were listed (Additional file 14: Table S9). The most upregulated genes were *Cttnbp2*, *Zfp608*, *SorbS2*, *Fam227a* and *St3gal*. The most downregulated genes were *Cd163*, *Padi2*, *Cth*, *Hsbp1l1* and *Nphs1*. Moreover, the GO and KEGG pathways were analyzed (Fig. 7b, c). Genes were enriched in the biological process of "metabolic process", the cellular components were enriched in "intracellular organelle" and the molecular functions were enriched in "ion binding". The most enriched KEGG process was "pyrimidine metabolism".

## Discussion

Loss of Langerhans' islet occurs in both type I and type II diabetes [42, 43]. Currently, islet transplantation is the main way to cure diabetes completely. However, the lack of available donor islets has prevented extensive use of this method [44]. Thus, identification of alternative islet sources may provide a gateway to widespread use of this practice to treat diabetes.

Recent evidence suggests that adult pancreatic progenitors may provide a potential source for beta-cell neogenesis in adults [2]. In rodents, ductal epithelium has been proven to be a potential progenitor pool in vivo [7]. Partial duct ligation [7] or treatment with exendin-4 [8] induced ductal cell proliferation and differentiation into beta cells, suggesting that insulin-producing cells can be generated from adult pancreatic ductal cells. Just like embryonic pancreatic progenitors, in-vitro cultured murine adult pancreatic ductal cells can reacquire self-renewal capacity and pluripotency [3, 11–13, 29]. These cultured ductal cells can differentiate into insulin-producing cells in vivo [10] and in vitro [9, 29]. Altogether, the adult pancreatic progenitors can serve as a promising beta-cell source for transplantation, although more research is needed.

Previously we reported that a 3D system was found to culture CD133^+^/SOX9^high^ pancreatic progenitor-like colonies [11]. This subpopulation could be expanded for many passages without losing its potential to differentiate to endocrine/acinar lineages. In addition, these cells can response to Wnt signal agonist R-spondin1. However, even if this is an excellent system to enrich these colonies, it is not perfect to study the regulation because of the conditioned medium which has uncertain components. Also, CD133^+^/SOX9^high^ were not proved to be all colony-forming units. To elucidate the mechanisms underlying the regulation of pancreatic progenitor cells, therefore, we modified the culturing system and used single cells from whole pancreas to generate pancreatic progenitor-like colonies and performed HTS to profile the transcriptome.

In our study, mouse pancreas was dissolved into single cells. In our modified system, these cells formed a cyst-like organoid. These colonies were PDX1^+^, CK7^+^, SOX9^+^ and weakly expressed NEUROG3. Also, they can differentiate to C-peptide secreting cells spontaneously. Following multiple passages, the cells did not lose self-renewal capacity and the ability to differentiate into other pancreatic lineages in a 2D system. Thus, our cultured pancreatic ductal epithelium exhibited pancreatic progenitor-like property and function.

To identify factors that regulate colony proliferation and differentiation, we profiled the colony cells’ transcriptome using HTS, a widely used technology to study the gene expression profile [45]. Total RNAs including mRNAs, miRNAs and lncRNAs are sequenced and annotated. As well as RNAs that have been discovered, new RNAs (mainly miRNAs and lncRNAs) can also be sequenced using this technology. By comparing the RNA expression profile between two samples, many candidate genes of differential expression can be revealed. In this study, total RNA of the colonies was extracted and sequenced, using whole pancreas as control. We captured 7266 significantly differentially expressed mRNAs along with 285 miRNAs and 183 lncRNAs. Expression of a subset of identified genes was confirmed by qPCR to verify HTS reliability. We found that CD133 was enriched in the colonies (Fig. 4b) and only CD133^+^ cells could form colonies (Fig. 5b). We also found that most of the cells in ring colonies were CD133^+^ (Fig. 5a) and had a similar gene expression pattern to CD133^+^ cell-derived colonies (Fig. 5c). Because CD133^+^ cells should represent all of the cells with the ability to form pancreatic progenitor-like colonies, this implied that our colonies were proper materials for HTS.

In addition, we noted that *Mettl10* is the most changed gene in upregulated mRNAs. METTL10 is a methyltransferase-like protein that trimethylates eukaryotic translation elongation factor 1 alpha 1(EF1A1), a eukaryotic elongation factor, which implies its contribution to many biological processes on a translational level [30]. This finding suggests that epigenetic regulation might play an important role in pancreatic progenitors. In order to find new surface markers, we verified quite a few cell surface proteins including signal pathway receptors, cell adhesion molecules and clusters of differentiation (CDs).

The miRNA showed an important role in beta-cell development [39]. A previous study showed that miR-21 regulates beta-cell death by targeting the tumor-suppressing gene *Pdcd4* [46]. Nevertheless, little is known of whether miRNA plays important roles in regulating pancreatic progenitors. In our results, miR-21a, miR-31, miR-200c and miR-155 were upregulated and miR-217, miR-802, miR-375 and miR-216 were downregulated (Additional file 9: Table S5). One differentially expressed miRNA of particular interest is miR-802, a transcriptional factor highly expressed in the liver and pancreas that targets HNF1B to regulate glycometabolism [38]. In addition, HNF1B is a crucial factor in pancreas development [47] and loss of HNF1B exhibits pancreas hypoplasia [48]. Also, HNF1B is upstream of SOX9 and NEUROG3 [48, 49], indicating its latent function to regulate pancreatic progenitors. In our study, *HNF1B* is upregulated in the colonies shown by HTS (log_2_ = 4.55, *p* < 0.05). Thus, miR-802 might be a potential miRNA regulating pancreatic progenitors. Another miRNA, miR-375, is a well-characterized miRNA regulating pancreas development [39]. miR-375 knockdown lead to reduced endocrine cells [50]. In conclusion, our profiling of the miRNA transcriptome provided a vast miRNA candidate pool for further research.

Our study also identified several differentially expressed lncRNAs, a group of noncoding RNAs which may regulate gene expression through various mechanisms [25]. Several lncRNAs play a role in pancreatic cancer [26], beta-cell biology [27] and pancreas development [28]. Recently a lncRNA was shown to regulate specification and function of beta cells by targeting multiple transcriptional factors [51]. Our profile identified 183 differentially expressed lncRNAs. In most upregulated lncRNAs, *Malat1* was shown to promote pancreatic cancer proliferation by stimulating autophagy [41], indicating its regulation effect on cell proliferation.

To reveal the functions of differentially expressed RNAs in our colonies, GO and KEGG analyses were performed. We note that the term "metabolic" was enriched in the biological process of both mRNAs and miRNAs. Also, in KEGG analysis of mRNA, the metabolic pathway was found to be significant. Metabolic pathways have been shown to have an important role in regulating stem cell functions [52]. Stemness is regulated by metabolic pathways in stem cells [53]. It is likely that pancreatic progenitors may share the same metabolic condition with other kinds of stem/progenitor cells. Regulation of metabolic pathways may also affect their functions.

## Conclusion

In summary, our study provides a system to enrich and culture pancreatic progenitor-like colonies in vitro, and HTS of the colonies should help understand the regulation of their proliferation and differentiation at a transcriptional level. HTS also offers a lot of candidate genes (including noncoding RNAs) which could be potential regulators or markers of pancreatic progenitors. By controlling these targets, we may manage to accumulate more colonies or force them to differentiate to beta lineage. In general, our results offer an efficient culture system for pancreatic progenitor-like colonies and the HTS of the colonies serves as a target resource for further study of in*-*vitro cultured pancreatic progenitors.

## Additional files

Additional file 1: is **Table S1** presenting primers used in RT-PCR and real-time PCR. (DOCX 15 kb)

Additional file 2: is **Figure S1** showing colony morphology and cell numbers in colonies. (**A**) Average-sized colonies photographed during 14 days of culture. *Scale bars*, 100 μm. (**B**) Colony size and forming frequency. Most colonies were less than 500 μm in diameter. (**C**) Cell numbers of different-sized colonies graphed with a fitted curve. (**D**) Colony-forming frequency in different basal medium. Data exhibited as the mean ± SD (*n* = 4). Two-tailed *t* test used to assess the differences. Significance defined as *p < 0.05, **p < 0.01 and ***p < 0.005. (**E**) After serial passaging, the colonies turned to be smaller (at P5). *Scale bars*, 200 μm. (PDF 1497 kb)

Additional file 3: is **Table S2** presenting statistics of the ring colonies in a 3D system with size and colony-forming frequency. (DOCX 13 kb)

Additional file 4: is **Figure S2** showing expression of the beta-lineage marker C-peptide. After 3 weeks of culture in a 3D system, some colonies crinkled to form a luminal structure. C-peptide could be detected at this stage. *Scale bars*, 50 μm. (PDF 1491 kb)

Additional file 5: is **Figure S3** showing cells from ring colonies could be induced to express multilineage markers. After 14 days of induction, RT-PCR confirmed the expression of *Ins1*, *Ins2* and other endocrine genes in clusters. Exocrine gene *Amylase* could also be detected in some clusters. (PDF 233 kb)

Additional file 6: is **Figure S4** showing differentially expressed lncRNAs detected by HTS and verified by real-time PCR. (**A**) Heat map of all lncRNA of the colonies and control. (**B**) Verification of differentially expressed lncRNAs. Data exhibited as the mean ± SD (*n* = 4). Two-tailed *t* test was used to assess the differences. Significance defined as *p < 0.05, **p < 0.01 and ***p < 0.005, comparison to the control group. (PDF 214 kb)

Additional file 7: is **Table S3** presenting the number of significantly changed RNAs between colonies and control. (DOCX 12 kb)

Additional file 8: is **Table S4** presenting top 25 differentially expressed mRNAs between colonies and control. (DOCX 14 kb)

Additional file 9: is **Table S5** presenting top 25 differentially expressed miRNAs between colonies and control. (DOCX 14 kb)

Additional file 10: is **Table S6** presenting top 25 differentially expressed lncRNAs between colonies and control. (DOCX 14 kb)

Additional file 11: is **Table S7** presenting original HTS data sets for miRNAs. (XLSX 172 kb)

Additional file 12: is **Table S8** presenting original HTS data sets for mRNAs and lncRNAs. (XLSX 12293 kb)

Additional file 13: is **Figure S5** showing GO and KEGG enrichment of lncRNAs. (**A**) GO enrichment of significantly changed lncRNA targets. Top 10 (by *p* value) of each GO dataset. *Numbers* in each part of the graph stand for the gene counts in each GO dataset. (**B**) Top 20 (by *p* value) of enriched KEGG pathways of lncRNA targets. *Numbers* on top of each column stand for the gene count in each KEGG pathway. (PDF 83 kb)

Additional file 14: is **Table S9** presenting top five overlapped genes and counts of targeting noncoding RNAs. (DOCX 13 kb)

## Abbreviations

BSA: Bovine serum albumin
Cela1: Chymotrypsin-like elastase family, member 1
Ck7: Cytokeratin 7
Ctrl: Chymotrypsin
Cyc: Cyclophillin
Dll1: Delta-like 1
EF1A1: Eukaryotic translation elongation factor 1 alpha 1
EGF: Epidermal growth factor
FBS: Fetal bovine serum
FCM: Flow cytometry
Fgfr2: Fibroblast growth factor receptor 2
Fzd1: Frizzled class receptor 1
GO: Gene Ontology
HNF1B: HNF1 homeobox B
HTS: High-throughput sequencing
Icam1: Intercellular adhesion molecule 1
KEGG: Kyoto Encyclopedia of Genes and Genomes
lncRNA: Long noncoding RNA
Mafa: v-maf musculoaponeurotic fibrosarcoma oncogene family, protein A
Malat1: Metastasis associated lung adenocarcinoma transcript 1
Mettl10: Methyltransferase like 10
miRNA: MicroRNA
Neurod1: Neurogenic differentiation 1
NEUROG3: Neurogenin 3
Nkx2.2: NK2 homeobox 2
Nkx6.1: NK6 homeobox 1
Pax4: Paired box 4
Pax6: Paired box 6
PCR: Polymerase chain reaction
Pdx1: Pancreatic and duodenal homeobox 1
Sox9: Sex determining region Y-box 9
TET: Ten–eleven translocation
Try4: Trypsin 4
