# Supplementary material for: Culturing and transcriptome profiling of progenitor-like colonies derived from adult mouse pancreas
Source: Stem Cell Res Ther. 2017 Jul 26;8:172. doi: 10.1186/s13287-017-0626-y (PMC5530554; doi:10.1186/s13287-017-0626-y)
Supplement: Supplementary file 1 — is Table S1 presenting primers used in RT-PCR and real-time PCR. (DOCX 15 kb) [file 13287_2017_626_MOESM1_ESM.docx]

| Gene Name | GenBank Accession | Forward Primer | Reverse Primer |
| --- | --- | --- | --- |
| *Ptf1a* | NM_018809.2 | TCCCATCCCCTTACTTTGATGA | GTAGCAGTATTCGTGTAGCTGG |
| *Amylase* | NM_001110505.1 | TCACACGGGTGATGTCAAGTT | GTCTGGGTTAATGCTCACTTCTT |
| *Sox9* | NM_011448.4 | CAGCCCCTTCAACCTTCCTC | TGATGGTCAGCGTAGTCGTATT |
| *Ck7* | NM_033073.3 | AGGAGATCAACCGACGCAC | GTCTCGTGAAGGGTCTTGAGG |
| *Nkx6.1* | NM_144955.2 | CTGCACAGTATGGCCGAGATG | CCGGGTTATGTGAGCCCAA |
| *Nkx2.2* | NM_001077632.1 | AAGCATTTCAAAACCGACGGA | CCTCAAATCCACAGATGACCAGA |
| *Mafa* | NM_194350.1 | AGGAGGAGGTCATCCGACTG | CTTCTCGCTCTCCAGAATGTG |
| *Pax4* | NM_001159925.1 | AGGGGGACTCTTTGTGAATGG | ACCTGTGCGGTAGTAGCGT |
| *Onecut1* | NM_008262.3 | GGCAACGTGAGCGGTAGTTT | TTGCTGGGAGTTGTGAATGCT |
| *Neurog3* | NM_009719.6 | CCAAGAGCGAGTTGGCACT | CGGGCCATAGAAGCTGTGG |
| *Pdx1* | NM_008814.3 | GAGGTGCTTACACAGCGGAA | GGGGCCGGGAGATGTATTT |
| *Glucagon* | NM_008100.4 | TTCCCAGAAGAAGTCGCCATT | GGTGCTCATCTCGTCAGAGAA |
| *Insulin1* | NM_008386.4 | CACTTCCTACCCCTGCTGG | ACCACAAAGATGCTGTTTGACA |
| *Insulin2* | NM_001185083.2 | GCAAGCAGGAAGGTTATTGTT | ACACACCAGGTAGAGAGCCT |
| *Pax6* | NM_001244198.2 | TACCAGTGTCTACCAGCCAAT | TGCACGAGTATGAGGAGGTCT |
| *Cyc* | NM_008907.1 | GAGCTGTTTGCAGACAAAGTTC | CCCTGGCACATGAATCCTGG |
| *Neurod1* | NM_010894.2 | ATGACCAAATCATACAGCGAGAG | TCTGCCTCGTGTTCCTCGT |
| *CD133* | NM_001163577.1 | ACTGAGAAATCCCCTACTGAAGT | GGCCTGTTTCGGCTTTCCTT |
| *Somatostatin* | NM_009215.1 | ACCGGGAAACAGGAACTGG | TTGCTGGGTTCGAGTTGGC |
| *Cela1* | NM_033612.2 | GTGGACACAGTACCGAGGAC | CCAGTTGCTTCGGATGAGGG |
| *Fgfr2* | NM_010207.2 | GCCTCTCGAACAGTATTCTCCT | ACAGGGTTCATAAGGCATGGG |
| *Cd44* | NM_001039150.1 | CCACAGCCTCCTTTCAATAACC | GGAGTCTTCGCTTGGGGTA |
| *Icam1* | NM_010493.3 | GTGATGCTCAGGTATCCATCCA | CACAGTTCTCAAAGCACAGCG |
| *Dll1* | NM_007865.3 | GACCTCGCAACAGAAAACCCA | TTCTCCGTAGTAGTGCTCGTC |
| *Cd38* | NM_007646.5 | TCTCTAGGAAAGCCCAGATCG | GTCCACACCAGGAGTGAGC |
| *Cd82* | NM_001136055.2 | TTCGGGGTGTGGATTCTTGC | AGGAAGCCCATCACTATGGTG |
| *Cd74* | NM_001042605.1 | AGTGCGACGAGAACGGTAAC | CGTTGGGGAACACACACCA |
| *Lepr* | NM_001122899.1 | GTCTTCGGGGTTGTGAATGTC | ACCTAAGGGTGGATCGGGTTT |
| *Cd93* | NM_010740.3 | ATCTCAACTGGTTTGTTCCTGC | ACTCTTCACGGTGGCAAGATT |
| *Pecam1* | NM_001032378.2 | GGTGCATGGCGTATCCAAG | TGGAGGTCTTATCTATCCTTCGC |

Tab. S1 Primers used in RT-PCR and Real-time PCR

Tab. S1 Primers used in RT-PCR and Real-time PCR. These primers were used to detect the expression of listed genes using RT-PCR or Real-time PCR essay.
