# Supplementary figures and images for: Culturing and transcriptome profiling of progenitor-like colonies derived from adult mouse pancreas
Source: Stem Cell Res Ther. 2017 Jul 26;8:172. doi: 10.1186/s13287-017-0626-y (PMC5530554; doi:10.1186/s13287-017-0626-y)

**A**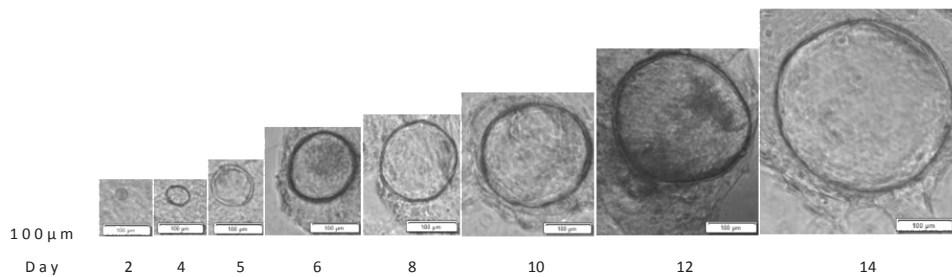**B**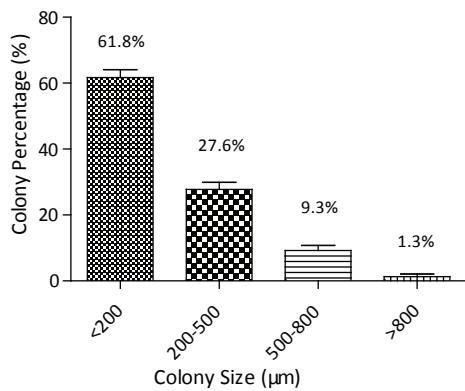**C**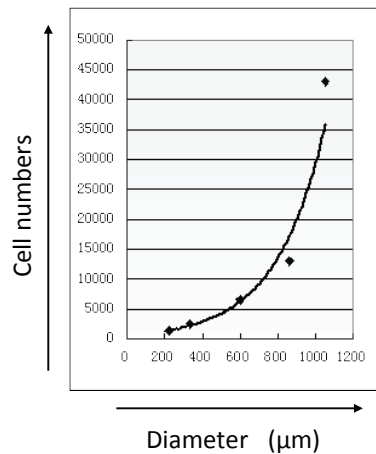**D**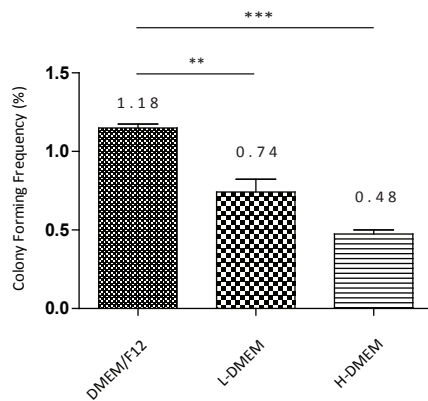**E**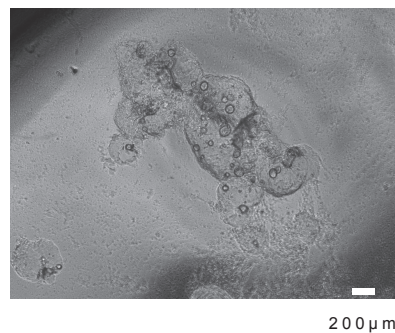

Supplement: Supplementary file 2 — is Figure S1 showing colony morphology and cell numbers in colonies. (A) Average-sized colonies photographed during 14 days of culture. Scale bars, 100 μm. (B) Colony size and forming frequency. Most colonies were less than 500 μm in diameter. (C) Cell numbers of different-sized colonies graphed with a fitted curve. (D) Colony-forming frequency in different basal medium. Data exhibited as the mean ± SD (n = 4). Two-tailed t test used to assess the differences. Significance defined as *p < 0.05, **p < 0.01 and ***p < 0.005. (E) After serial passaging, the colonies turned to be smaller (at P5). Scale bars, 200 μm. (PDF 1497 kb) [file 13287_2017_626_MOESM2_ESM.pdf]

A

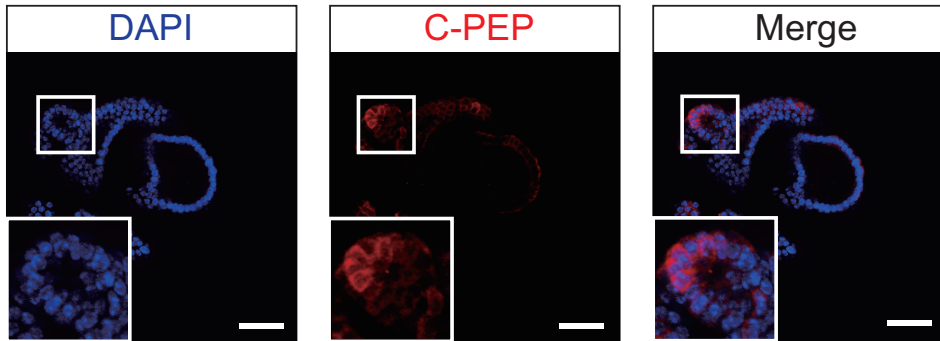

Supplement: Supplementary file 4 — is Figure S2 showing expression of the beta-lineage marker C-peptide. After 3 weeks of culture in a 3D system, some colonies crinkled to form a luminal structure. C-peptide could be detected at this stage. Scale bars, 50 μm. (PDF 1491 kb) [file 13287_2017_626_MOESM4_ESM.pdf]

**A**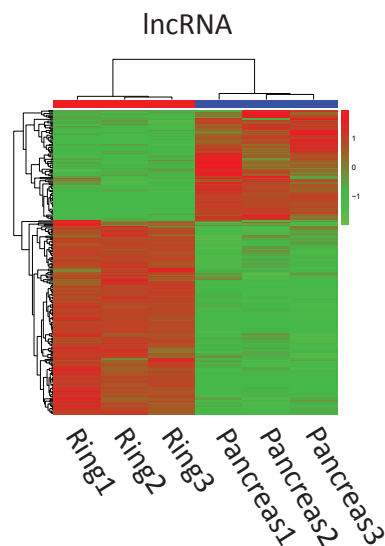**B**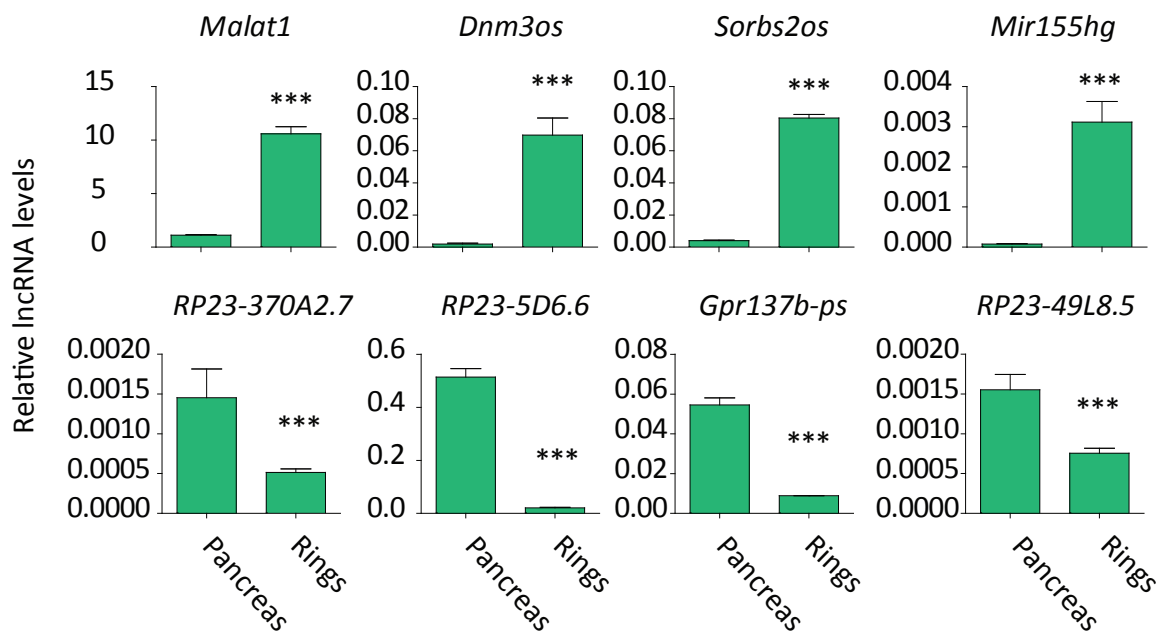

Supplement: Supplementary file 6 — is Figure S4 showing differentially expressed lncRNAs detected by HTS and verified by real-time PCR. (A) Heat map of all lncRNA of the colonies and control. (B) Verification of differentially expressed lncRNAs. Data exhibited as the mean ± SD (n = 4). Two-tailed t test was used to assess the differences. Significance defined as *p < 0.05, **p < 0.01 and ***p < 0.005, comparison to the control group. (PDF 214 kb) [file 13287_2017_626_MOESM6_ESM.pdf]

**A**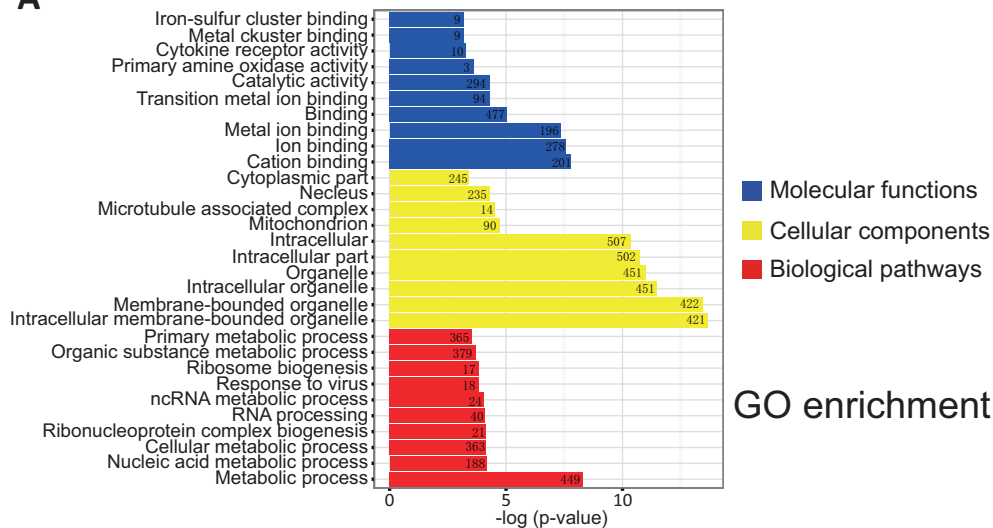**B**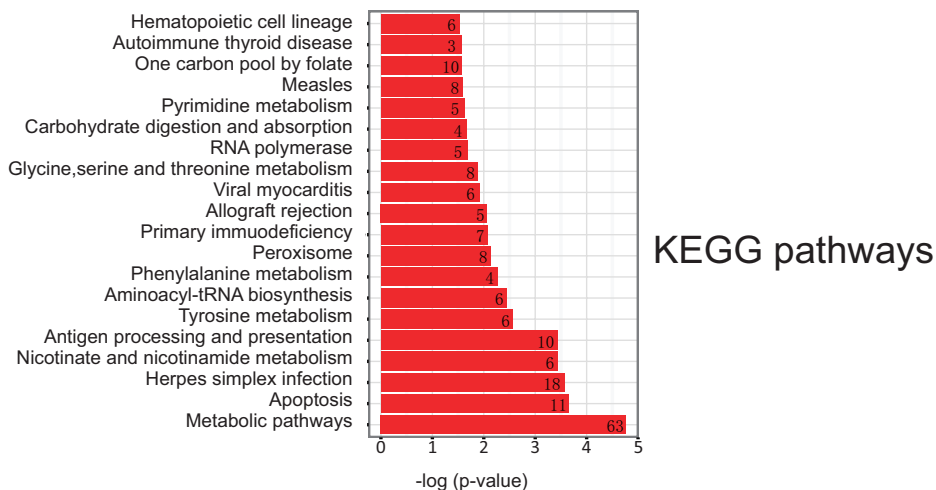

Supplement: Supplementary file 13 — is Figure S5 showing GO and KEGG enrichment of lncRNAs. (A) GO enrichment of significantly changed lncRNA targets. Top 10 (by p value) of each GO dataset. Numbers in each part of the graph stand for the gene counts in each GO dataset. (B) Top 20 (by p value) of enriched KEGG pathways of lncRNA targets. Numbers on top of each column stand for the gene count in each KEGG pathway. (PDF 83 kb) [file 13287_2017_626_MOESM13_ESM.pdf]
