# Supplementary material for: Culturing and transcriptome profiling of progenitor-like colonies derived from adult mouse pancreas
Source: Stem Cell Res Ther. 2017 Jul 26;8:172. doi: 10.1186/s13287-017-0626-y (PMC5530554; doi:10.1186/s13287-017-0626-y)
Supplement: Supplementary file 3 — is Table S2 presenting statistics of the ring colonies in a 3D system with size and colony-forming frequency. (DOCX 13 kb) [file 13287_2017_626_MOESM3_ESM.docx]

TableS2. Statistics of the ring colonies in 3D system with size and colony-forming frequency.

| Well | 1 | 2 | 3 | 4 | Average |
| --- | --- | --- | --- | --- | --- |
| <200μm | 61 | 56 | 66 | 50 | 58.3 |
| 200-500μm | 22 | 27 | 26 | 29 | 26.0 |
| 500-800μm | 13 | 7 | 6 | 9 | 8.8 |
| >800μm | 3 | 0 | 0 | 2 | 1.3 |
| Total | 99 | 90 | 98 | 90 | 94.3±4.9±4.9 |
| Colony-forming frequency | 1.23% | 1.12% | 1.23% | 1.13% | 1.18%±0.06% |

TableS2. Statistics of the ring colonies in 3D system with size and colony-forming frequency. 8000 cells were cultured in one well of a 24-well plate in 3D system for 2 weeks. Total colonies in each well (n=4) were counted under an convert microscope.
