# Supplementary material for: Culturing and transcriptome profiling of progenitor-like colonies derived from adult mouse pancreas
Source: Stem Cell Res Ther. 2017 Jul 26;8:172. doi: 10.1186/s13287-017-0626-y (PMC5530554; doi:10.1186/s13287-017-0626-y)
Supplement: Supplementary file 5 — is Figure S3 showing cells from ring colonies could be induced to express multilineage markers. After 14 days of induction, RT-PCR confirmed the expression of Ins1, Ins2 and other endocrine genes in clusters. Exocrine gene Amylase could also be detected in some clusters. (PDF 233 kb) [file 13287_2017_626_MOESM5_ESM.pdf]

A

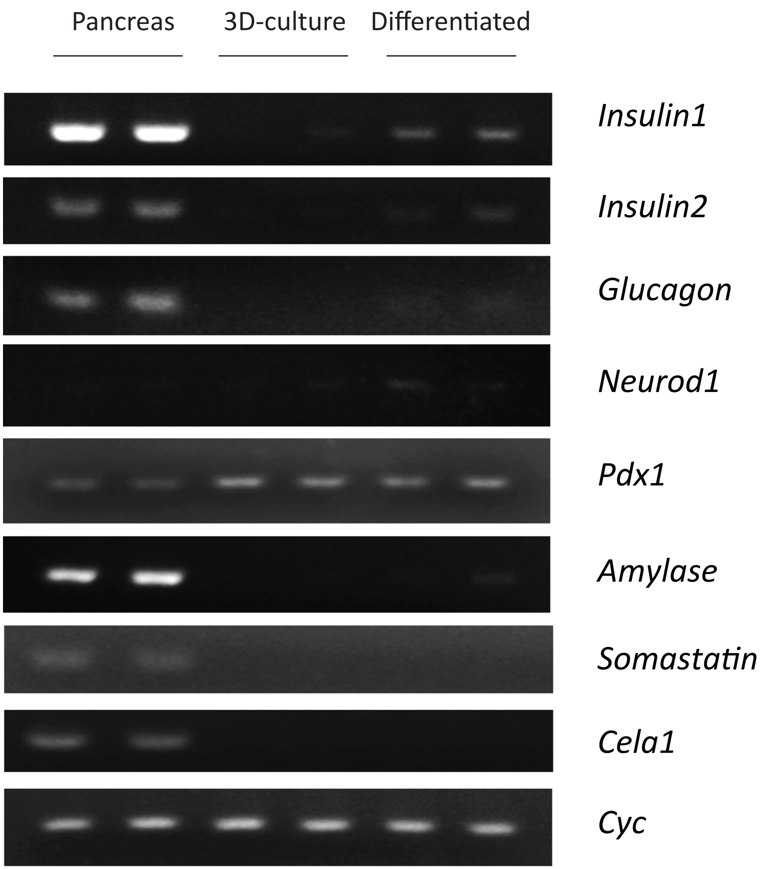

Pancreas
3D-culture
Differentiated
Insulin1
Insulin2
Glucagon
Neurod1
Pdx1
Amylase
Somastatin
Cela1
Cyc
