# Supplementary material for: Culturing and transcriptome profiling of progenitor-like colonies derived from adult mouse pancreas
Source: Stem Cell Res Ther. 2017 Jul 26;8:172. doi: 10.1186/s13287-017-0626-y (PMC5530554; doi:10.1186/s13287-017-0626-y)
Supplement: Supplementary file 7 — is Table S3 presenting the number of significantly changed RNAs between colonies and control. (DOCX 12 kb) [file 13287_2017_626_MOESM7_ESM.docx]

TableS3. The number of significantly changed RNAs between the colonies and control.

|  | Significant RNA change in the colonies | | |
| --- | --- | --- | --- |
|  | Up-regulated | Down-regulated | Total |
| mRNA | 3564 | 3702 | 7266 |
| miRNA | 251 | 34 | 285 |
| lncRNA | 119 | 64 | 183 |

TableS3. The number of significantly changed RNAs between the colonies and control. Three independent samples of colonies and their pre-culture controls (pancreas) were harvested for HTS. Total significant RNA change was collected.
