# Supplementary material for: Culturing and transcriptome profiling of progenitor-like colonies derived from adult mouse pancreas
Source: Stem Cell Res Ther. 2017 Jul 26;8:172. doi: 10.1186/s13287-017-0626-y (PMC5530554; doi:10.1186/s13287-017-0626-y)
Supplement: Supplementary file 8 — is Table S4 presenting top 25 differentially expressed mRNAs between colonies and control. (DOCX 14 kb) [file 13287_2017_626_MOESM8_ESM.docx]

TableS4. Top25 of differentially expressed mRNAs between the colonies and control.

| **Up-regulated**  **mRNAs**  **(n=3564)** | **Fold Change**  **(log2)** | **Down-regulated mRNAs**  **（n=3702）** | **Fold Change**  **(log2)** |
| --- | --- | --- | --- |
| Mettl10 | 1044.11 | Try4 | -18.60 |
| Zcchc14 | 646.95 | Gm24357 | -18.33 |
| Fgfr2 | 279.34 | Gm24299 | -18.32 |
| Gm10800 | 12.50 | Ctrl | -18.08 |
| Mmp7 | 10.27 | Cela3b | -17.80 |
| Sftpd | 9.56 | Prss2 | -17.14 |
| Onecut2(Variant1) | 8.91 | 2210010C04Rik | -17.13 |
| Cldn2 | 8.49 | Try5 | -17.08 |
| Col12a1 | 8.15 | Cpa1 | -16.46 |
| Saa3 | 7.88 | Rnase1 | -16.30 |
| Onecut2(Variant2) | 7.82 | Pnliprp1 | -16.17 |
| Lcn2 | 7.75 | Pnliprp2 | -15.79 |
| Cxcl5 | 7.74 | Clps | -15.65 |
| Gabrp | 7.40 | Cela1 | -15.59 |
| Cp | 7.31 | Gp2 | -15.57 |
| Inhbb | 6.87 | Ctrb1 | -14.87 |
| Lox | 6.72 | Spink1 | -13.18 |
| Dusp4 | 6.69 | Cckar | -12.20 |
| Cp(Variant1) | 6.68 | Gatm | -12.08 |
| Mmp3 | 6.66 | Ins1 | -11.22 |
| Col28a1 | 6.56 | Try10 | -10.71 |
| Slc4a4 | 6.52 | Bhlha15 | -9.99 |
| Kif27 | 6.48 | Tff2 | -9.87 |
| Serpine1 | 6.44 | Prss3 | -9.84 |
| Cp(Variant2) | 6.39 | Gm5771 | -9.38 |

TableS4. Top25 of differentially expressed mRNAs between the colonies and control by HTS. Fold change was showed in log2.
