# Supplementary material for: Culturing and transcriptome profiling of progenitor-like colonies derived from adult mouse pancreas
Source: Stem Cell Res Ther. 2017 Jul 26;8:172. doi: 10.1186/s13287-017-0626-y (PMC5530554; doi:10.1186/s13287-017-0626-y)
Supplement: Supplementary file 9 — is Table S5 presenting top 25 differentially expressed miRNAs between colonies and control. (DOCX 14 kb) [file 13287_2017_626_MOESM9_ESM.docx]

TableS5. Top25 of differentially expressed miRNAs between the colonies and control.

| **Up-regulated**  **MicroRNAs**  **(n=251)** | **Fold Change**  **(log2)** | **Down-regulated**  **MicroRNAs**  **（n=34）** | **Fold Change**  **(log2)** |
| --- | --- | --- | --- |
| mmu-miR-21a-3p | 10.50 | mmu-miR-216b-3p | -10.96 |
| mmu-miR-31-5p | 10.19 | mmu-miR-802-3p | -9.13 |
| mmu-miR-3473e | 9.16 | mmu-miR-217-5p | -9.10 |
| mmu-miR-21c | 8.95 | mmu-miR-216a-3p | -9.02 |
| mmu-let-7i-3p | 8.91 | mmu-miR-216b-5p | -8.96 |
| mmu-miR-210-3p | 8.78 | mmu-miR-6969-3p | -8.72 |
| mmu-miR-29b-3p | 8.50 | mmu-miR-216a-5p | -7.24 |
| mmu-miR-22-3p | 8.32 | mmu-miR-802-5p | -6.81 |
| mmu-miR-3473b | 8.13 | mmu-miR-375-3p | -6.66 |
| mmu-miR-130a-3p | 8.12 | mmu-miR-184-3p | -4.97 |
| mmu-miR-708-5p | 8.03 | mmu-miR-148a-3p | -4.94 |
| mmu-miR-206-3p | 8.01 | mmu-miR-7b-5p | -4.05 |
| mmu-miR-34c-3p | 7.96 | mmu-miR-153-3p | -3.95 |
| mmu-miR-21a-5p | 7.89 | mmu-miR-126b-5p | -3.29 |
| mmu-miR-155-5p | 7.80 | mmu-miR-325-5p | -2.58 |
| mmu-miR-106b-5p | 7.68 | mmu-miR-451a | -2.56 |
| mmu-miR-31-3p | 7.61 | mmu-miR-144-5p | -2.50 |
| mmu-miR-125b-1-3p | 7.57 | mmu-miR-129-1-3p | -2.46 |
| mmu-miR-425-3p | 7.55 | mmu-miR-204-5p | -2.42 |
| mmu-miR-92a-1-5p | 7.37 | mmu-miR-148a-5p | -2.31 |
| mmu-miR-34b-5p | 7.37 | mmu-miR-150-5p | -2.10 |
| mmu-miR-1247-3p | 7.36 | mmu-miR-338-5p | -2.00 |
| mmu-miR-205-5p | 7.25 | mmu-miR-384-5p | -2.00 |
| mmu-miR-200c-5p | 7.08 | mmu-miR-6538 | -1.87 |
| mmu-miR-298-5p | 7.01 | mmu-miR-1a-3p | -1.80 |

TableS5. Top25 of differentially expressed miRNAs between the colonies and control by HTS. Fold change was showed in log2.
