# Supplementary material for: Culturing and transcriptome profiling of progenitor-like colonies derived from adult mouse pancreas
Source: Stem Cell Res Ther. 2017 Jul 26;8:172. doi: 10.1186/s13287-017-0626-y (PMC5530554; doi:10.1186/s13287-017-0626-y)
Supplement: Supplementary file 10 — is Table S6 presenting top 25 differentially expressed lncRNAs between colonies and control. (DOCX 14 kb) [file 13287_2017_626_MOESM10_ESM.docx]

TableS6. Top25 of differentially expressed lncRNAs between the colonies and control.

| **Up-regulated**  **lncRNAs**  **(n=112)** | **Fold Change**  **(log2)** | **Down-regulated lncRNAs**  **（n=51）** | **Fold Change**  **(log2)** |
| --- | --- | --- | --- |
| Gm26870 | 10.74 | Gm43525 | -6.09 |
| Gm26870 | 9.16 | Gpr137b-ps | -5.73 |
| Malat1 | 6.96 | E230001N04Rik | -5.54 |
| A530020G20Rik | 5.81 | 1810008I18Rik | -4.93 |
| 4732490B19Rik | 5.34 | AW112010 | -4.64 |
| Dnm3os | 4.40 | Gm26580 | -4.19 |
| Sorbs2os | 4.40 | Gm15063 | -4.09 |
| Mir155hg | 4.21 | Sardhos | -3.71 |
| Dnm3os | 3.97 | Gm33051 | -3.54 |
| Gm26742 | 3.96 | 4933404O12Rik | -3.52 |
| Gm12576 | 3.67 | RP23-49L8.5 | -3.45 |
| Gm26772 | 3.65 | 1810063I02Rik | -3.40 |
| Gm20186 | 3.64 | 1810008I18Rik | -3.36 |
| Gm15567 | 3.55 | RP23-370A2.7 | -3.33 |
| Gm26809 | 3.47 | Gm17035 | -3.29 |
| Gm42428 | 3.14 | Gm17024 | -3.23 |
| Gm26669 | 3.13 | D730003I15Rik | -3.15 |
| Hoxb3os | 3.11 | 2310040G24Rik | -2.86 |
| Kirrel3os | 3.09 | Gm9725 | -2.74 |
| Gm26568 | 3.05 | RP23-5D6.6 | -2.73 |
| Gm26889 | 3.01 | Gm11963 | -2.73 |
| D130017N08Rik | 2.90 | Gm12953 | -2.71 |
| RP23-259J8.9 | 2.90 | B230334C09Rik | -2.68 |
| Gm26786 | 2.88 | 2310010J17Rik | -2.64 |
| Egfros | 2.77 | 1700018L02Rik | -2.64 |

TableS6. Top25 of differentially expressed lncRNAs between the colonies and control by HTS. Fold change was showed in log2.
