# Supplementary material for: Culturing and transcriptome profiling of progenitor-like colonies derived from adult mouse pancreas
Source: Stem Cell Res Ther. 2017 Jul 26;8:172. doi: 10.1186/s13287-017-0626-y (PMC5530554; doi:10.1186/s13287-017-0626-y)
Supplement: Supplementary file 14 — is Table S9 presenting top five overlapped genes and counts of targeting noncoding RNAs. (DOCX 13 kb) [file 13287_2017_626_MOESM14_ESM.docx]

| mRNA | Fold change | Targeting miRNA counts | | Targeting lncRNA counts | |
| --- | --- | --- | --- | --- | --- |
|  |  | Up-regulated | Down-regulated | Up-regulated | Down-regulated |
| Cttnbp2 | 4.89516 | 173 | 35 | 1 | 0 |
| St3gal5 | 4.65557 | 30 | 9 | 1 | 0 |
| Fam227a | 4.48872 | 110 | 23 | 2 | 0 |
| Sorbs2 | 4.179115 | 63 | 21 | 1 | 0 |
| Zfo608 | 3.73782 | 22 | 2 | 1 | 0 |
| Nphs1 | -9.78828 | 24 | 8 | 0 | 3 |
| Hsbp1l1 | -7.31797 | 12 | 5 | 0 | 2 |
| Cth | -7.2824 | 34 | 12 | 0 | 1 |
| Padi2 | -6..3967 | 82 | 16 | 5 | 4 |
| Cd163 | -6.11929 | 8 | 8 | 0 | 1 |

TableS9. Top5 of overlapped genes and the counts of targeting non-coding RNAs.

TableS9. Top5 of overlapped genes and the counts of targeting non-coding RNAs. To find key mRNA regulated by non-coding RNA network, we performed co-expression analysis of differentially expressed RNAs between the colonies and control. The intersection of significantly changed mRNAs and the potential targets of all significantly changed non-coding RNAs were obtained. We noted 304 overlapped genes. The most up-regulated genes were Cttnbp2, Zfp608, SorbS2, Fam227a and St3gal. The most down-regulated genes were Cd163, Padi2, Cth, Hsbp1l1 and Nphs1.
